# Depression Mediates the Association Between Ambient Air Pollution and Gastrointestinal/Liver Diseases: A Prospective Cohort Study

**DOI:** 10.1002/cns.70878

**Published:** 2026-04-16

**Authors:** Yanqi Kou, Shicai Ye, Mouji Liang, Lei Ge, Ling Qin, Yanping Ha, Yuan Tian, Botao Luo, Chunyi Wu, Liping Zhan, Ke Yang, Zhuoyan Lu, Yuping Yang

**Affiliations:** ^1^ Department of Gastroenterology Affiliated Hospital of Guangdong Medical University, Guangdong Medical University Zhanjiang Guangdong China; ^2^ Department of Gastrointestinal Surgery Affiliated Hospital of Guangdong Medical University, Guangdong Medical University Zhanjiang Guangdong China; ^3^ Department of Hematology The First Affiliated Hospital and College of Clinical Medicine of Henan University of Science and Technology Luoyang Henan China; ^4^ Department of Pathology Guangdong Medical University Zhanjiang China

**Keywords:** air pollution, depression, gastrointestinal diseases, liver diseases, public health

## Abstract

**Background:**

The escalating burden of gastrointestinal (GI) and liver diseases poses a critical public health challenge, with emerging evidence implicating air pollution as a modifiable risk factor. However, the mechanistic pathways, particularly the mediating role of depression in the pollution‐disease nexus, remain underexplored. This study investigates the longitudinal associations between ambient air pollution exposure and GI/liver diseases in a nationwide cohort, with a focus on quantifying depression's mediating effects.

**Methods:**

Utilizing data from the China Health and Retirement Longitudinal Study (CHARLS, 2011–2020; *n* = 18,755), we evaluated the impact of long‐term exposure to PM_1_, PM_2.5_, PM_10_, SO_2_, CO, NO_2_, and O_3_ on incident GI/liver diseases. Multivariable‐adjusted Cox proportional hazards models incorporated demographic, socioeconomic (region, education, retirement status), and lifestyle (smoking, drinking) covariates. Mediation analysis assessed the proportion of total effects mediated by depression (CESD‐10 scores).

**Results:**

Chronic exposure to PM_1_ (HR = 1.33, 95% CI: 1.30–1.37), PM_2.5_ (HR = 1.30, 1.26–1.33), PM_10_ (HR = 1.29, 1.25–1.32), SO_2_ (HR = 1.64, 1.60–1.69), CO (HR = 1.47, 1.43–1.51), and NO_2_ (HR = 1.27, 1.23–1.30) per interquartile range increase was significantly associated with elevated disease risk, whereas O_3_ exhibited protective effects (HR = 0.77, 0.74–0.79). Depression mediated 4.0%–11.3% of pollution effects, with the highest mediation observed for O_3_ (11.3%) and lowest for SO_2_ (4.0%). Stratified analyses revealed heightened vulnerability in rural residents, individuals with lower education, and Northeastern populations.

**Conclusions:**

This study pioneers the identification of depression as a mediator linking air pollution to GI/liver diseases in a nationally representative cohort. The findings advocate for integrative policies targeting air quality improvement and mental health interventions to alleviate the dual burden of environmental and psychological morbidity.

## Graphical abstract

This prospective cohort study of 18,755 Chinese adults reveals that long‐term exposure to ambient air pollutants is associated with increased risk of gastrointestinal and liver diseases. Depression symptoms significantly mediate these associations. The findings underscore the necessity for integrated public health policies that concurrently address air pollution and mental health.

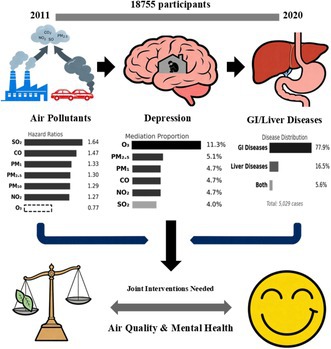

## 1 Introduction

The global incidence of gastrointestinal (GI) and liver diseases has surged dramatically, emerging as a leading contributor to morbidity and mortality worldwide. Epidemiological estimates attribute approximately 2 million annual deaths to these conditions, representing 4% of global mortality—a burden that persists despite intensified preventive efforts [1, 2]. Alarmingly, cirrhosis and chronic liver pathologies alone caused 1.26 million deaths in 2019, reflecting a 13% increase since 1990 [2, 3]. These diseases consistently rank among the top ten causes of mortality, imposing unsustainable strains on healthcare systems and underscoring the urgent need to address modifiable environmental determinants [4, 5].

Concurrently, air pollution has been recognized as a systemic health threat with far‐reaching pathophysiological and socioeconomic ramifications [6, 7]. Beyond its established role in respiratory and cardiovascular pathologies, chronic exposure to airborne pollutants exacerbates neurodegenerative processes, elevates all‐cause mortality, and induces cognitive decline [8]. Emerging spatial epidemiological data further reveal robust associations between particulate matter (PM: PM_1_, PM_2.5_, PM_10_) and gaseous pollutants (SO_2_, CO, NO_2_) with metabolic dysregulation, including diabetes, dyslipidemia, and hypertension [9, 10, 11]. Mechanistic evidence corroborates direct hepatotoxic and enterotoxic effects of PM through oxidative stress, microbiota disruption, and epigenetic modifications, while multicenter cohorts demonstrate heightened risks of inflammatory bowel disease (IBD) and cirrhosis in polluted regions [12, 13, 14, 15, 16, 17].

Notably, depression—affecting 280 million individuals globally—exhibits bidirectional relationships with somatic diseases. With prevalence rates escalating to 5.7% in aging populations, depression amplifies susceptibility to infections (40%–60% increased sepsis risk) and coexists with 34.7% of IBD/cirrhosis cases [18, 19, 20, 21, 22, 23]. Critically, over 30% of depressed patients present concurrent GI comorbidities, suggesting intricate gut‐brain‐liver axis interactions that may potentiate environmental toxicity [24].

Despite evidence linking air pollution to GI/hepatic pathologies, critical gaps persist in understanding depression's mediating role within this association. This study leverages longitudinal data from the China Health and Retirement Longitudinal Study (CHARLS; *n* = 18,755) to: (1) quantify the effects of PM_1_, PM_2.5_, PM_10_, SO_2_, CO, NO_2_, and O_3_ on incident GI/liver diseases; and (2) elucidate the proportion of pollution‐disease effects mediated by depression, thereby informing integrated intervention strategies. To validate the generalizability of our primary findings and examine whether associations between air pollution and digestive diseases observed in the CHARLS cohort are consistent with global epidemiological patterns, we conducted a secondary ecological correlation analysis using data from the Global Burden of Disease (GBD) 2023 study. This complementary analysis leverages standardized, internationally comparable estimates of disease burden and environmental exposure across 195 countries/regions.

## 2 Methods

### 2.1 Study Population and Participant Selection

**FIGURE 1:**
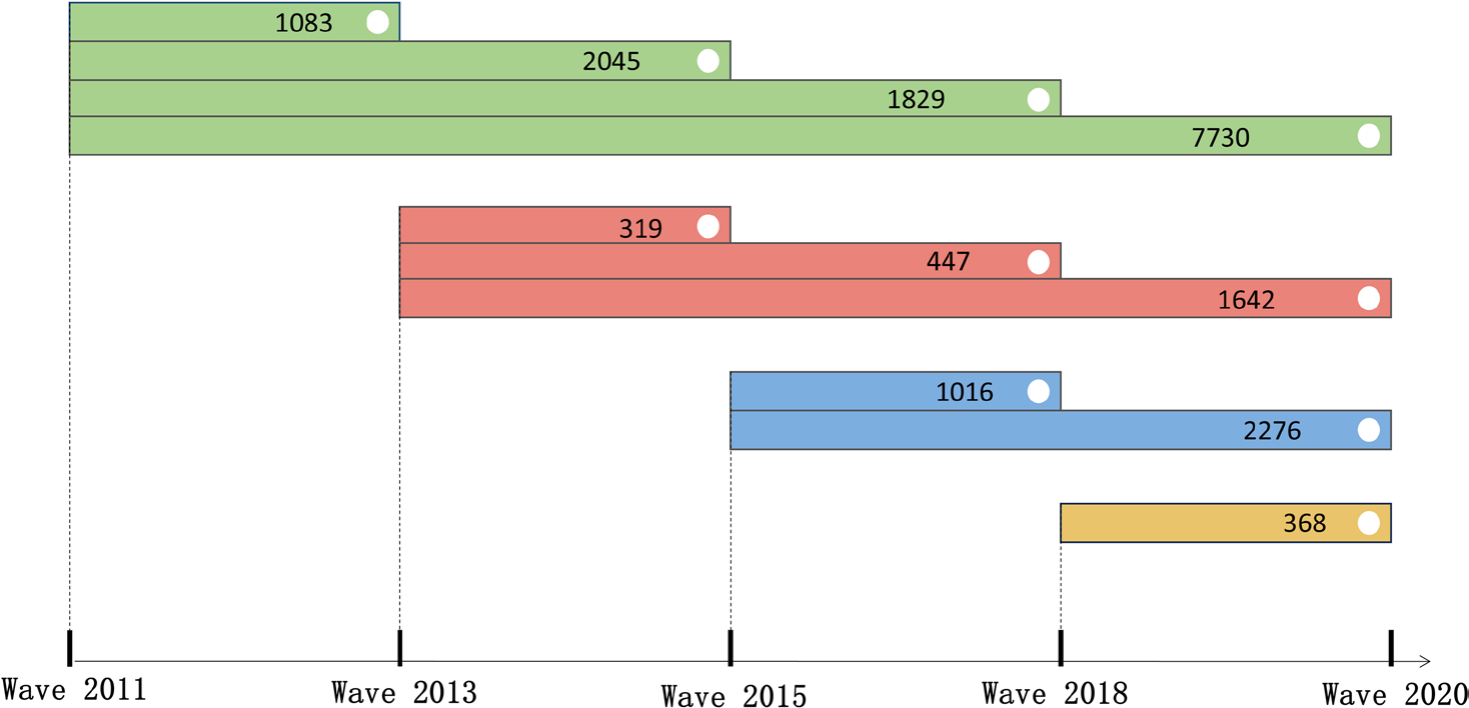
Study participant flowchart.

The study population was derived from the CHARLS, a nationwide cohort representing 28 provinces in China. The CHARLS study protocol was approved by the Ethics Review Board of Peking University (IRB00001052‐11015) and adhered to the principles outlined in the Declaration of Helsinki [25]. Informed consent was obtained from all participants prior to their involvement in the study. The study began with 17,708 participants in 2011. After excluding non‐participants, those with unknown disease status, and those with pre‐existing GI or liver diseases, 12,687 participants remained. Follow‐up surveys in 2013, 2015, 2018, and 2020 included the following participants: 1083 in 2013, 2045 in 2015, 1829 in 2018, and 7730 in 2020. In 2013, 3426 new participants were included, and after applying exclusion criteria, 2408 remained. In 2015, 3824 participants were enrolled, and 3292 were eligible after exclusions. In 2018, 628 new participants were included, with 368 eligible participants remaining after exclusions. By the end of the 2020 wave, the total number of participants was 18,755. Figure 1 illustrates the participant selection process, showing the number of participants at each wave and the exclusions that occurred, leading to the final cohort for analysis.

### 2.2 Data Collection and Outcome Assessment

Data were collected on demographic, socioeconomic, lifestyle, and health‐related factors to assess the relationship between air pollution and GI and liver diseases. Demographic variables included age, sex, body mass index (BMI), and residence type (urban or rural). Socioeconomic variables included region (Eastern, Central, Western, or Northeast), education level (elementary school or below, middle school, or higher), marital status (married or unmarried), and retirement status (No, Yes). Lifestyle factors, such as physical activity (PA), smoking, and drinking consumption, were recorded as binary variables (yes or no). Health‐related variables included self‐reported health status (good/very good, fair, or poor/very poor). Depression was assessed using the CESD‐10 score, which was included as a covariate to examine its mediating role in the relationship between air pollution and GI and liver diseases.

GI and liver diseases were diagnosed based on responses to health questionnaires in baseline and follow‐up surveys, asking whether participants had been diagnosed by a doctor with digestive or liver diseases (excluding cancer and tumors). Those who answered “Yes” were classified as having these diseases.

### 2.3 Air Pollution Exposure Assessment

Air pollution data were obtained from the China High‐Resolution, High‐Quality Near Surface Air Pollutant dataset, which integrates multiple data sources, including ground‐based measurements, satellite remote sensing products, atmospheric reanalysis outputs, and model simulations [26]. The dataset has a spatial resolution of 0.1° (approximately 10 km) and provides accurate estimations of air pollution concentrations, including PM_1_, PM_2.5_, PM_10_, SO_2_, NO_2_, CO, and O_3_, based on participants' residential geocoded locations. These geocoded locations were matched with artificial intelligence algorithms to compute ground‐level concentrations of pollutants across the study area. To assess air pollution exposure, the exposure levels of the pollutants were averaged for each participant from the time of cohort entry until the occurrence of GI or liver diseases for those who developed the diseases. For participants who did not develop these diseases, the exposure was averaged from cohort entry until the end of the study in 2020.

### 2.4 Validation Analysis: Air Pollution and Digestive Diseases Using GBD 2023 Data

To assess the generalizability of our primary findings at a global population level, we conducted an ecological correlation analysis using data from the GBD 2023 study. This analysis aimed to explore the cross‐national associations between major air pollutants and digestive diseases. We extracted age‐standardized incidence and/or prevalence rates (per 100,000 population) for three major digestive disease categories from the GBD 2023 results: liver cirrhosis, IBD, and upper digestive diseases. These categories were selected because they represent the major digestive disease classifications in GBD with standardized definitions across countries, and they directly correspond to or overlap with the liver and GI disease outcomes in our primary CHARLS analysis. Concurrently, we obtained three key air pollutants with comprehensive global estimates: PM, NO_2_, and O_3_. These three pollutants were selected because they are the only air pollutants with comprehensive global estimates in GBD 2023. We employed Generalized Additive Models (GAMs) to model the potentially non‐linear relationships between each pollutant and each disease.

### 2.5 Statistical Analyses

To address the issue of missing data, this study employed multiple imputation. The exposure and outcome data in this study were complete. Missingness was present in several covariates, with the proportions detailed in Table S1. Data analysis was performed using R software (version 4.2.2). Descriptive statistics were calculated for baseline characteristics, where continuous variables were presented as mean ± standard deviation (SD), and categorical variables as frequencies (percentages). The associations between air pollution exposure and GI and liver diseases were examined using Cox proportional hazards models. Initially, the models were constructed without any adjustments (Model 1). In the adjusted models (Model 2 and Model 3), the analyses were controlled for age, sex, region, residence type, marital status, education level, BMI, smoking, drinking, health status, and PA. Hazard ratios (HRs) and 95% confidence intervals (CIs) for each pollutant were calculated per interquartile range (IQR) increment in pollutant concentrations. To assess the potential confounding due to correlations among pollutants and to examine the independent effect of each pollutant, we further conducted two‐pollutant model analyses. Specifically, for each target air pollutant, we fitted additional Cox proportional hazards models that included the target pollutant and one other pollutant at a time. This approach allowed us to evaluate the stability of the association for each pollutant when adjusting for a potential co‐pollutant. Prior to modeling, we examined inter‐pollutant correlations using Spearman correlation coefficients to identify potential multicollinearity.

Restricted cubic spline (RCS) analysis was employed to investigate the potential nonlinear relationships between air pollution exposure and the risk of GI and liver diseases. The RCS model allowed for the examination of nonlinear effects, providing insight into the risk changes across different levels of exposure. Subgroup and interaction analyses were conducted to examine how the associations between air pollution and GI and liver diseases varied across different demographic and health‐related variables, including sex, residence type, region, education level, marital status, smoking, drinking, and health status. Mediation analyses were performed to examine the role of depression (measured by the CESD‐10 score) as a mediator in the relationship between air pollution and GI and liver diseases. Indirect and direct effects were estimated for each pollutant, and the proportions mediated by depression were calculated. To address temporal coherence concerns, we performed a sensitivity analysis using the pre‐event CESD‐10 score (closest to outcome occurrence) rather than baseline score, and confirmed the robustness of mediation estimates across both temporal strategies. The analysis employed the standard causal mediation framework without exposure–mediator interaction terms, using the “mediation” package in R version 4.2.2. A Quasi‐Bayesian approach with 1000 simulations was used to estimate direct and indirect effects, with 95% confidence intervals derived from the percentile method of the simulation distribution.

Sensitivity analyses were performed to assess the robustness of the findings. First, continuous variables of air pollution exposure were used to reanalyze the associations between air pollutants and GI and liver diseases. Second, participants with poor or very poor health were excluded from the analysis to evaluate whether the results remained consistent. Third, propensity score matching (PSM) was used to match participants with GI and liver diseases to an equal number without these diseases based on demographic characteristics, using a 1:1 nearest‐neighbor ratio without replacement and a caliper width of 0.05. Post‐PSM results were compared to pre‐PSM findings to confirm the robustness of the associations. In addition, GI and liver diseases were analyzed as independent endpoint events. All statistical analyses were performed using R software (version 4.2.2), with significance defined as a two‐tailed *p*‐value of less than 0.05.

## 3 Results

### 3.1 Characteristics of Study Participants

**FIGURE 2:**
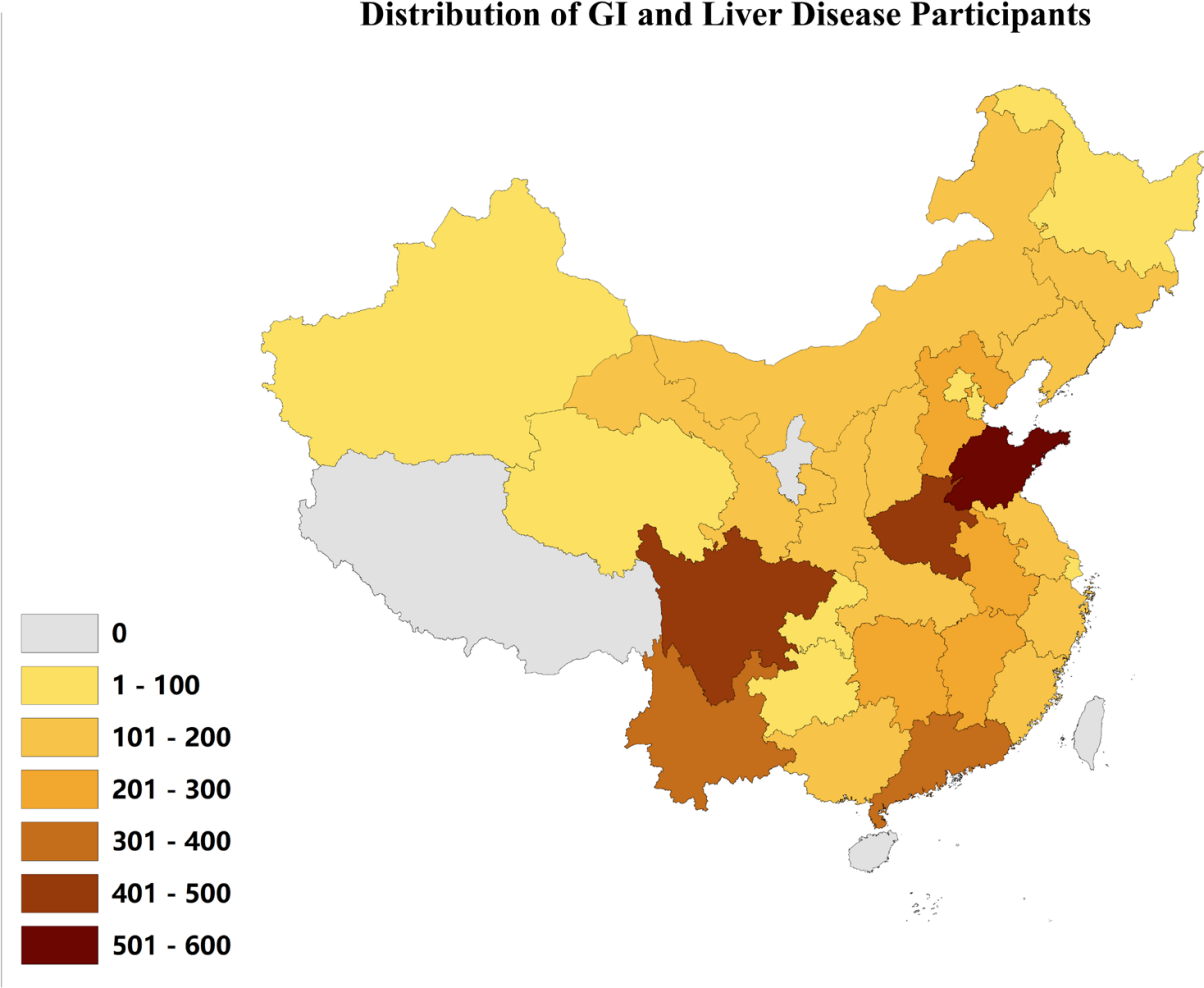
Geographic distribution of participants with GI and liver diseases.

The analytical cohort comprised 18,755 participants (mean age: 56 ± 10 years), stratified into two groups: individuals diagnosed with GI/liver diseases (*n* = 5029) and disease‐free controls (*n* = 13,726). Key demographic and clinical characteristics are summarized in Table 1 and visualized geographically in Figure 2.

**TABLE 1:** Basic characteristics of participants.

Characteristics	Total (*n* = 18,755)	Non‐GI and liver diseases (*n* = 13,726)	GI and liver diseases (*n* = 5029)	*p*
Age (years, *M* ± SD)	56 ± 10	57 ± 11	56 ± 10	0.003
BMI (kg/m^2^, *M* ± SD)	24.0 ± 3.8	24.0 ± 3.7	23.9 ± 4.0	< 0.001
CESD‐10 (*M* ± SD)	8 ± 6	7 ± 6	10 ± 6	< 0.001
Region, *n* (%)				0.453
Northeast	1393 (7.4%)	1037 (7.6%)	356 (7.1%)	
Eastern	8579 (45.7%)	6252 (45.5%)	2327 (46.3%)	
Central	2809 (15.0%)	2039 (14.9%)	770 (15.3%)	
Western	5974 (31.9%)	4398 (32.0%)	1576 (31.3%)	
Disease, *n* (%)				< 0.001
Liver	832 (4.4%)	0 (0.0%)	832 (16.5%)	
GI	3917 (20.9%)	0 (0.0%)	3917 (77.9%)	
GI and liver	280 (1.5%)	0 (0.0%)	280 (5.6%)	
Sex, *n* (%)				< 0.001
Female	9163 (48.9%)	6880 (50.1%)	2283 (45.4%)	
Male	9592 (51.1%)	6846 (49.9%)	2746 (54.6%)	
Residence, *n* (%)				< 0.001
Urban	8111 (43.2%)	6109 (44.5%)	2002 (39.8%)	
Rural	10,644 (56.8%)	7617 (55.5%)	3027 (60.2%)	
Marital status, *n* (%)				0.138
Single	2002 (10.7%)	1493 (10.9%)	509 (10.1%)	
Married	16,753 (89.3%)	12,233 (89.1%)	4520 (89.9%)	
Health status, *n* (%)				< 0.001
Poor/very poor	4079 (21.7%)	2555 (18.6%)	1524 (30.3%)	
Fair	9259 (49.4%)	6717 (48.9%)	2542 (50.5%)	
Good/very good	5417 (28.9%)	4454 (32.4%)	963 (19.1%)	
Smoking, *n* (%)				0.004
No	11,434 (61.0%)	8282 (60.3%)	3152 (62.7%)	
Yes	7321 (39.0%)	5444 (39.7%)	1877 (37.3%)	
Drinking, *n* (%)				0.167
No	10,582 (56.4%)	7703 (56.1%)	2879 (57.2%)	
Yes	8173 (43.6%)	6023 (43.9%)	2150 (42.8%)	
Education level, *n* (%)				< 0.001
Elementary school or below	12,942 (69.0%)	9301 (67.8%)	3641 (72.4%)	
Middle school or above	5813 (31.0%)	4425 (32.2%)	1388 (27.6%)	
PA, *n* (%)				0.251
No	1891 (10.61%)	1404 (10.77%)	487 (10.17%)	
Yes	15,935 (89.39%)	11,634 (89.23%)	4301 (89.83%)	
Retirement status, *n* (%)				0.009
No	16,551 (88.2%)	12,062 (87.9%)	4489 (89.3%)	
Yes	2204 (11.8%)	1664 (12.1%)	540 (10.7%)	

Abbreviations: BMI, body mass index (calculated as weight in kilograms divided by height in meters squared); CESD‐10, Center for Epidemiological Studies Depression Scale‐10; GI, gastrointestinal; PA, physical activity.

*Group Comparisons: Age and BMI*: The disease group exhibited a younger mean age (56 ± 10 vs. 57 ± 11 years; *p* = 0.003) and lower BMI (23.9 ± 4.0 vs. 24.0 ± 3.7 kg/m^2^; *p* < 0.001). Depression: CESD‐10 scores were markedly elevated in the disease group (10 ± 6 vs. 7 ± 6; *p* < 0.001). Residence: A higher proportion of cases resided in rural areas (60.2% vs. 55.5%; *p* < 0.001), though regional distribution (Eastern: 45.7%, Western: 31.9%, Central: 15.0%, Northeast: 7.4%) did not differ significantly between groups (*p* = 0.453). Health Status: Poor/very poor self‐rated health was more prevalent among cases (30.3% vs. 18.6%; *p* < 0.001). Lifestyle: Current smoking rates were higher in the non‐disease group (39.7% vs. 37.3%; *p* = 0.004), while no significant differences emerged in marital status, alcohol consumption, or PA (*p* > 0.05 for all).

### 3.2 Associations Between Air Pollution and GI and Liver Diseases

Chronic exposure to air pollutants demonstrated robust associations with GI and liver diseases across analytical models (Table 2). In the unadjusted model (Model 1), all pollutants—including PM_1_ (HR = 1.31, 95% CI: 1.28–1.34), PM_2.5_ (HR = 1.29, 1.26–1.33), PM_10_ (HR = 1.29, 1.26–1.32), SO_2_ (HR = 1.56, 1.52–1.61), CO (HR = 1.46, 1.43–1.50), NO_2_ (HR = 1.23, 1.20–1.26), and O_3_ (HR = 0.86, 0.84–0.88)—showed statistically significant associations (*p* < 0.001 for all).

**TABLE 2:** Associations between air pollution and GI and liver diseases.

Air pollutants	Model 1			Model 2			Model 3		
	HR	95% CI	*p*	HR	95% CI	*P*	HR	95% CI	*p*
PM_1_	1.31	1.28, 1.34	< 0.001	1.31	1.27, 1.34	< 0.001	1.33	1.30, 1.37	< 0.001
PM_2.5_	1.29	1.26, 1.33	< 0.001	1.29	1.26, 1.32	< 0.001	1.30	1.26, 1.33	< 0.001
PM_10_	1.29	1.26, 1.32	< 0.001	1.28	1.25, 1.32	< 0.001	1.29	1.25, 1.32	< 0.001
SO_2_	1.56	1.52, 1.61	< 0.001	1.56	1.52, 1.60	< 0.001	1.64	1.60, 1.69	< 0.001
CO	1.46	1.43, 1.50	< 0.001	1.46	1.42, 1.50	< 0.001	1.47	1.43, 1.51	< 0.001
O_3_	0.86	0.84, 0.88	< 0.001	0.85	0.83, 0.87	< 0.001	0.77	0.74, 0.79	< 0.001
NO_2_	1.23	1.20, 1.26	< 0.001	1.23	1.19, 1.26	< 0.001	1.27	1.23, 1.30	< 0.001

*Note:* Model 1: no covariates were adjusted. Model 2: adjusted for sex and age. Model 3: adjusted for region, sex, age, residence, marital status, health status, BMI, smoking, drinking, education level, retirement status, and PA.

Abbreviations: CI, confidence interval; CO, carbon monoxide; HR, hazard ratio; NO_2_, nitrogen dioxide; O_3_, ozone; PM_1_, particle with aerodynamic diameter ≤ 1 μm; PM_10_, particle with aerodynamic diameter ≤ 10 μm; PM_2.5_, particle with aerodynamic diameter ≤ 2.5 μm; SO_2_, sulfur dioxide.

Adjustment for age and sex (Model 2) attenuated HR marginally but retained significance for all pollutants (*p* < 0.001). In the fully adjusted model (Model 3; incorporating socioeconomic, lifestyle, and health covariates), PM_1_ (HR = 1.33, 95% CI: 1.30–1.37), PM_2.5_ (HR = 1.30, 1.26–1.33), PM_10_ (HR = 1.29, 1.25–1.32), SO_2_ (HR = 1.64, 1.60–1.69), CO (HR = 1.47, 1.43–1.51), and NO_2_ (HR = 1.27, 1.23–1.30) exhibited dose‐dependent risks IQR increment (*p* < 0.001). Notably, O_3_ displayed a pronounced inverse association in Model 3 (HR = 0.77, 0.74–0.79; *p* < 0.001), suggesting potential protective effects against GI/liver pathologies. The Spearman correlations among all seven air pollutants are presented in Table S2. Most pollutants showed moderate to high positive correlations with each other. Notably, O_3_ was positively correlated with NO_2_ (*r* = 0.62, *p* < 0.001). The results from the two‐pollutant models are detailed in Table S3. The inverse association between O_3_ and GI/liver diseases remained significant and was strengthened after adjustment for each of the other pollutants. For example, when adjusted for NO_2_, the HR for O_3_ per IQR increase was 0.51 (95% CI: 0.49–0.53; *p* < 0.001). In contrast, the associations for certain particulate matters (e.g., PM_1_ and PM_2.5_) were attenuated when mutually adjusted, indicating the influence of correlated exposures.

These results underscore the differential impacts of pollutant classes: particulate matter (PM_1_, PM_2.5_, PM_10_) and gaseous pollutants (SO_2_, CO, NO_2_) consistently elevated disease risk, whereas O_3_ emerged as a potential mitigating factor. The paradoxical protective role of O_3_ warrants mechanistic investigation to disentangle its anti‐inflammatory properties from confounding environmental interactions.

### 3.3 Nonlinear Associations Between Air Pollution and GI and Liver Diseases

**FIGURE 3:**
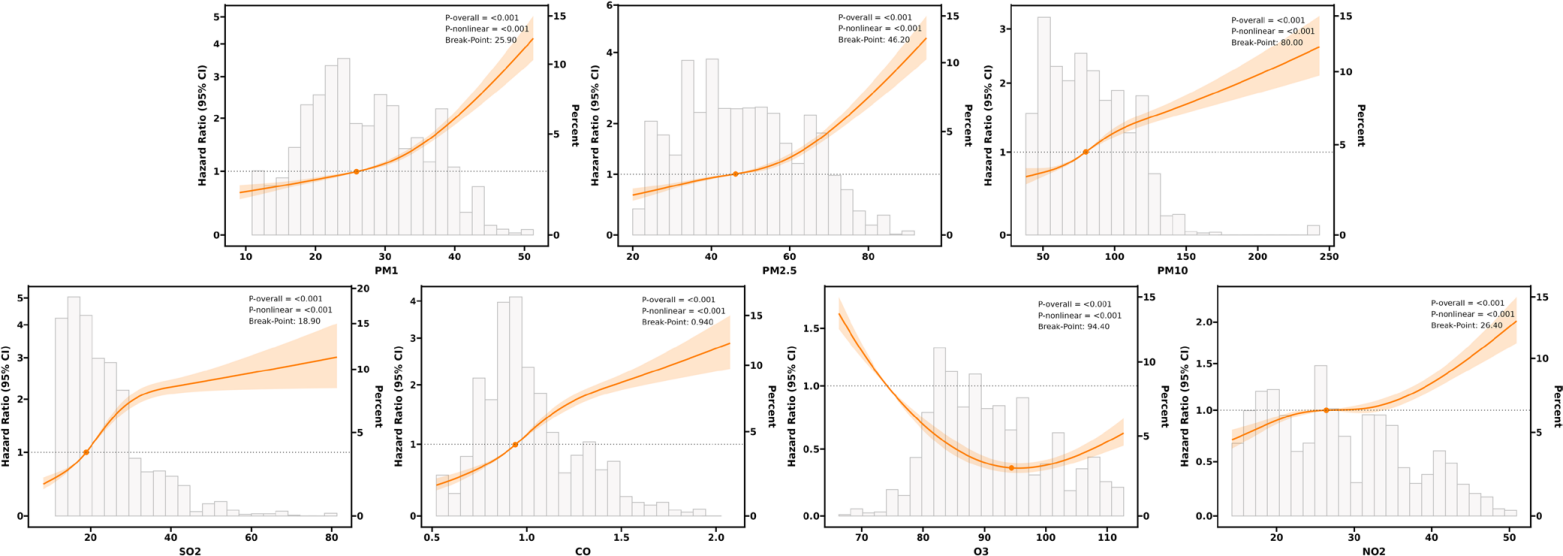
Nonlinear relationship between air pollution exposure and GI and liver disease risk.

RCS analysis revealed significant nonlinear exposure‐response relationships between air pollution levels and GI/liver disease risk (*P* for nonlinearity < 0.001; Figure 3). While disease risk exhibited a monotonic increase with rising pollutant concentrations, distinct exposure‐response patterns emerged: PM_1_ demonstrated a J‐shaped curve relationship within 10–50 μg/m^3^, with a critical threshold at 25.90 μg/m^3^; PM_2.5_ showed markedly elevated risk in the 60–80 μg/m^3^ range, peaking at a threshold of 46.20 μg/m^3^; PM_10_ displayed a continuously ascending risk trend with an inflection point at 80.00 μg/m^3^. Among gaseous pollutants, O_3_ exhibited a U‐shaped association with HR < 1 in the 76–110 μg/m^3^ range, whereas NO_2_ presented a sustained upward risk trend beyond its threshold of 26.40 μg/m^3^. SO_2_ demonstrated a toxicity threshold at 18.90 μg/m^3^, and CO induced a steep risk escalation within 0.5–1.5 mg/m^3^. These findings collectively indicate threshold‐dependent toxicity mechanisms, where pollutant effects transition from moderate to accelerated harm upon reaching critical exposure levels.

The identification of nonlinear and threshold dynamics underscores the inadequacy of linear models for risk estimation and highlights the need for pollutant‐specific regulatory benchmarks in environmental health policymaking.

### 3.4 Subgroup Analysis of the Associations Between Air Pollution and GI and Liver Diseases

**FIGURE 4:**
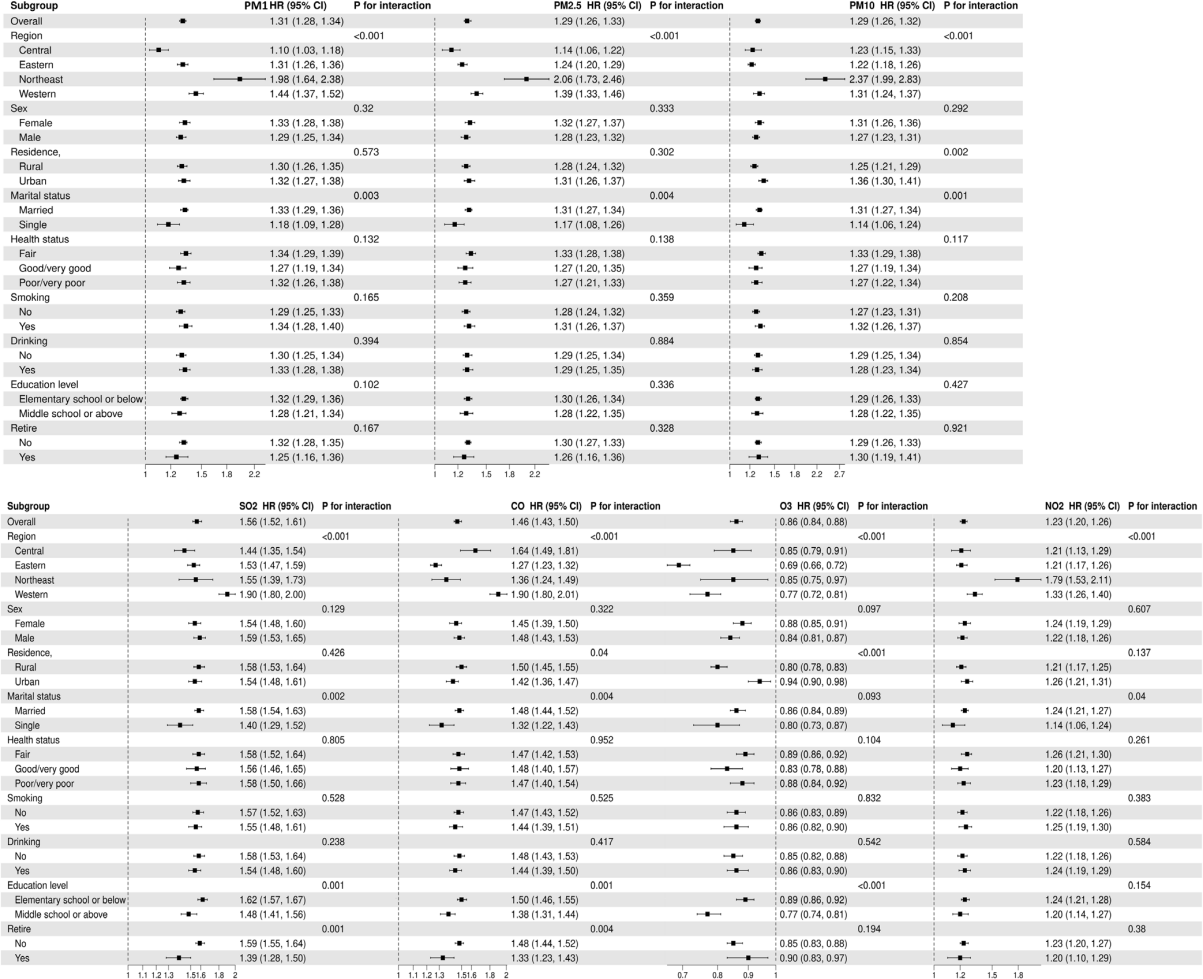
Subgroup analysis of air pollution and GI and liver diseases.

Subgroup analyses revealed marked heterogeneity in pollution‐disease associations across demographic strata (*p* for interaction < 0.05 for key variables; Figure 4).

Regional Disparities:Northeast China: Exhibited the strongest associations, particularly for PM_1_ (HR = 1.98, 95% CI: 1.64–2.38), PM_2.5_ (HR = 2.06, 1.73–2.46), PM_10_ (HR = 2.37, 1.99–2.83), and NO_2_ (HR = 1.79, 1.53–2.11) (*p* < 0.001 for all), aligning with its elevated industrial/coal‐derived pollution levels. Western China: Dominated by SO_2_ (HR = 1.90, 1.80–2.00) and CO (HR = 1.90, 1.80–2.01) risks (*p* < 0.001), consistent with biomass combustion and mining activities. Central/Eastern China: Demonstrated weaker but significant PM‐related risks (e.g., PM_1_ HR = 1.10, 1.03–1.18; PM_2.5_ HR = 1.14, 1.06–1.22, *p* < 0.001 for all). O_3_ showed protective effects in Central (HR = 0.85, 0.79–0.91) and Northeast regions (*p* < 0.001).

Demographic Interactions: Residence Type: Urban populations faced higher PM_10_ (HR = 1.36, 1.30–1.41; *p* = 0.002) and O_3_ risks (HR = 0.94, 0.90–0.98; *p* < 0.001), whereas rural residents exhibited elevated CO susceptibility (HR = 1.50, 1.45–1.55; *p* = 0.04). Socioeconomic Status: Lower education amplified risks from SO_2_ (HR = 1.62, 1.57–1.67; *p* = 0.001), CO (HR = 1.50, 1.46–1.55; *p* = 0.001), and O_3_ (HR = 0.89, 0.86–0.92; *p* < 0.001). Employment Status: Nonretired individuals had higher SO_2_ (HR = 1.59, 1.55–1.64; *p* = 0.001) and CO (HR = 1.48, 1.44–1.52; *p* = 0.004) risks, likely reflecting occupational/commuting exposures. Marital Status: Married participants showed heightened vulnerability to pollution effects (*p* < 0.05). No significant interactions were observed for gender, health status, smoking, or alcohol use (*p* > 0.05).

These stratified analyses identify high‐risk subgroups—rural dwellers, less‐educated individuals, and non‐retired workers—as priority targets for pollution mitigation strategies. Regional heterogeneity underscores the need for location‐specific interventions tailored to dominant pollutant profiles (e.g., PM/NO_2_ control in Northeast China, SO_2_/CO reduction in Western China).

### 3.5 Mediating Role of Depression in the Association Between Air Pollution and GI and Liver Diseases

Mediation analyses quantified the role of depression in linking air pollution to GI/liver diseases. Initial models confirmed a dose–response relationship between CESD‐10 scores and disease risk: each 1‐unit increase in continuous CESD‐10 scores elevated risk by 6% (HR = 1.06, 95% CI: 1.06–1.06; *p* < 0.001), with categorical analyses showing a 1.4‐fold risk escalation in participants with severe depression (CESD‐10 ≥ 11) versus non‐depressed counterparts (Table S4).

**FIGURE 5:**
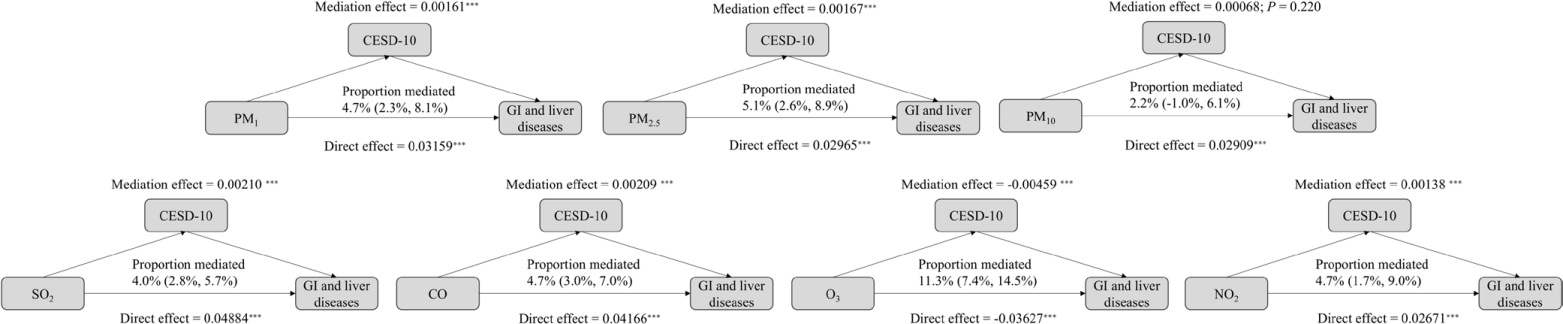
Mediation analysis of depression in the relationship between air pollution and GI and liver diseases.

Formal mediation testing revealed depression mediated 4.0%–11.3% of pollution effects (*p* < 0.05 for all except PM_10_ [*p* = 0.220]; Figure 5, Table S5): Highest mediation: O_3_ (11.3%, 95% CI: 7.4%–14.5%); Lowest mediation: SO_2_ (4.0%, 95% CI: 2.8%–5.7%); Intermediate effects: PM_1_ (4.7%), PM_2.5_ (5.1%), CO (4.7%), NO_2_ (4.7%). To assess the robustness of our mediation estimates to temporal assumptions about mediator measurement, we repeated the analysis using the most recent pre‐event CESD‐10 score rather than baseline CESD‐10. Results are presented in Table S6. The mediation proportions remained remarkably consistent, indicating robustness to different temporal measurement strategies.

These results establish depression as both an independent risk factor and a partial mediator in pollution‐disease pathways. Notably, O_3_'s paradoxical association—protective overall yet partially mediated by depression—suggests its dual role as an environmental oxidant and neuroactive agent.

### 3.6 Sensitivity Analysis of the Association Between Air Pollution and GI and Liver Diseases

Sensitivity analyses confirmed the robustness of the associations between air pollution and GI and liver diseases. PSM was performed to control for potential confounders. After matching 4970 participants with GI and liver diseases to an equal number without, baseline characteristics between the two groups showed no significant differences (Table S7). Post‐PSM results aligned closely with pre‐PSM findings, further supporting the robustness of the associations (Table S8). When continuous variables of air pollution were used in the analysis instead of categorizing pollutants by IQR increments, the significant associations for all pollutants remained consistent (Table S9). Further sensitivity analyses excluding participants with poor or very poor health did not substantially alter the results, with effect sizes and statistical significance remaining similar to the main findings for all pollutants, including O_3_ (Table S10). To address potential heterogeneity between GI and liver diseases, we analyzed these endpoints separately (Tables S11 and S12). Overall, the direction of associations was consistent, but effect sizes differed. To address potential concerns regarding the handling of missing data, an additional sensitivity analysis was conducted using the original, unimputed dataset (complete‐case analysis). The results of this analysis for the association between air pollutants and GI/liver diseases are presented in Table S13. The hazard ratios and confidence intervals derived from this complete‐case analysis were highly consistent with the primary results obtained from the multiply imputed data, reinforcing the robustness of our core findings to the method of missing data handling.

Overall, these sensitivity analyses demonstrated that the associations between various air pollutants (PM_1_, PM_2.5_, SO_2_, CO, O_3_, and NO_2_) and the prevalence of GI and liver diseases were robust across different analytical approaches, including the use of continuous exposure variables and adjustments for confounders.

### 3.7 Validation Study: Associations Between Air Pollution and Digestive Diseases Using GBD 2023 Data

To assess the generalizability of our primary findings at the population level, an ecological correlation analysis was conducted using data from the GBD 2023 study. The associations between three major air pollutants (PM, NO_2_, and O_3_) and the incidence and prevalence of liver cirrhosis, IBD, and upper GI diseases were examined (Figure S1).

Significant positive correlations were observed between the incidence and prevalence of liver cirrhosis and PM, NO_2_, and O_3_ pollution (*p* < 0.001 or *p* < 0.05). For IBD, the incidence and prevalence showed a plateauing association slope with PM and O_3_ pollution, with no significant correlation (*p* > 0.05); however, a moderate positive correlation trend followed by a slight decline was observed with NO_2_ pollution (*p* < 0.001). The incidence and prevalence of upper GI diseases exhibited an overall increasing trend followed by a plateau and slight decline in association with PM and NO_2_ pollution (*p* < 0.001), while the association slope with O_3_ pollution appeared to plateau, showing no statistically significant association (*p* > 0.05).

These ecological findings provide external, population‐level support for the adverse associations between PM and NO_2_ and digestive diseases observed in our cohort. The disease‐specific relationships, particularly the distinct association pattern for O_3_, highlight the complexity of air pollution effects on different digestive organs and warrant further investigation.

## 4 Discussion

This nationwide cohort study provides novel evidence that long‐term exposure to ambient air pollutants—particularly particulate matter (PM_1_, PM_2.5_, PM_10_) and gaseous pollutants (SO_2_, CO, NO_2_, O_3_)—significantly elevates the incidence of GI and liver diseases in China, with depression mediating 4.0%–11.3% of these effects. Our findings advance current understanding of environmental hepatogastroenterology by establishing three key insights: air pollution exhibits pollutant‐ and region‐specific toxicity profiles; depression operates as both an independent risk factor and a mediator in pollution‐disease pathways; and O_3_ displays paradoxical protective effects, challenging conventional paradigms of pollutant toxicity.

### 4.1 Contextualizing Air Pollution‐Disease Associations

Our results align with global evidence linking air pollution to GI/hepatic pathologies but extend prior work through granular exposure‐response quantification. The utilization of household solid fuels, which is strongly associated with heightened indoor concentrations of PM_2.5_ and CO, has been epidemiologically established as a significant contributor to increased disease burden [27, 28, 29]. Li et al. reported that chronic PM_2.5_, PM_10_, and NO_2_ exposure significantly elevates risks of peptic ulcers and chronic gastritis [30, 31, 32]. Our prior investigations have consistently demonstrated an association between air pollution exposure and elevated GI disease risk among Chinese adults [12, 33]. Hepatic studies similarly implicate air pollutants in nonalcoholic fatty liver diseases, particularly through oxidative stress and metabolic dysregulation [34, 35, 36, 37]. Zhan et al. demonstrated that PM_2.5_, PM_10_, and NO_2_ alter DNA methylation patterns in GI risk genes (e.g., *IL21R*, *EVPL*), enhancing disease susceptibility [38]. Barouki et al. demonstrated that PM_2.5_ undergoes systemic translocation, subsequently inducing hepatic oxidative stress, inflammatory responses, and Kupffer cell activation. Concurrently, it disrupts intestinal microbiota homeostasis, thereby facilitating endotoxin translocation and promoting hepatic fibrogenesis [39, 40, 41]. The observed inverse association between O_3_ exposure and GI/liver diseases in this study warrants further discussion. Notably, O_3_ uniquely displayed protective effects against GI and liver diseases, consistent with its anti‐inflammatory and antioxidant properties [12, 42, 43]. Recent epidemiological research has found that higher O_3_ concentrations were associated with a 12% and 11% reduction in the incidence of NAFLD and advanced liver fibrosis, respectively [44]. Mechanistically, experimental studies indicate that controlled O_3_ exposure can activate the antioxidant defense system by enhancing catalase activity and reducing levels of the oxidative stress biomarker TBARS, while simultaneously suppressing pro‐inflammatory cytokines, including (TNF‐α), and elevating anti‐inflammatory markers such as IL‐10 [45, 46]. Furthermore, animal studies have demonstrated that O_3_ treatment alleviates hepatic edema, necrosis, and leukocyte infiltration, accompanied by improvements in liver function markers (ALT, AST, GGT, bilirubin, and albumin) [47]. These antioxidant and anti‐inflammatory effects may explain the inverse correlation observed in our longitudinal analysis. The results represent a preliminary epidemiological observation, not a causal claim. They nonetheless indicate that O_3_ may exert complex biological effects, pointing to a new research direction regarding environmental oxidants and digestive health.

### 4.2 Depression as a Mechanistic Linchpin

Our mediation analyses reveal a previously under characterized pathway: air pollution induces neuroinflammation and oxidative stress, thereby promoting depression, which subsequently drives the development of GI and hepatic pathology [48]. This cascade likely operates through microbiota‐gut‐liver‐brain axis disruption [49, 50, 51, 52], where pollutants and depressive states synergistically impair intestinal permeability, amplify hepatic inflammation, and dysregulate serotonin metabolism [53, 54, 55]. Notably, the mediation proportion for O_3_ (11.3%—highest among pollutants) implies its unique capacity to modulate both oxidative damage and mood pathways, potentially via NMDA receptor interactions [54]. These findings necessitate a paradigm shift toward integrated mental‐environmental health interventions.

### 4.3 Regional and Demographic Vulnerabilities

The stark regional disparities—Northeast China's PM/NO_2_ dominance versus Western China's SO_2_/CO predominance—mirror spatial pollution source distributions: industrialized regions suffer combustion‐related PM [56, 57, 58], while rural areas face biomass‐derived CO [59]. Socioeconomic gradients further compound risks; lower education amplified SO_2_ susceptibility by 62%, likely reflecting limited access to air filtration and healthcare [60, 61, 62]. These patterns underscore the urgency for region‐tailored mitigation strategies, such as transitioning Northeast China from coal‐based heating to renewable energy and subsidizing air purifiers in Western rural households.

### 4.4 Innovation and Limitations

This study pioneers the integration of mediation analysis into environmental hepatogastroenterology, quantitatively linking depression to pollution‐disease pathways—a critical advance beyond prior correlative reports. However, three limitations merit consideration: Exposure Misclassification: Residential pollutant estimates may not capture occupational/mobility‐based exposures, potentially attenuating effect sizes. Self‐Report Bias: Physician‐diagnosed outcomes, while widely used in CHARLS, lack the precision of endoscopic/histological verification. Generalizability: Single‐country designs necessitate validation in populations with distinct pollution profiles. Additionally, the absence of detailed dietary data at baseline in CHARLS precluded direct adjustment for dietary factors. We partially addressed potential confounding from lifestyle and diet by adjusting for correlated proxies (BMI, alcohol use, smoking, education, region, and urbanicity) and by including PA. Although residual confounding due to unmeasured dietary factors cannot be entirely ruled out, the stability of estimates across models suggests that our primary conclusions are unlikely to be driven solely by omitted lifestyle variables. Future studies incorporating validated dietary assessments will help refine these estimates. Although temporal ordering between exposure and depression cannot be definitively established from baseline data, the robustness of our mediation estimates to different temporal assumptions about mediator measurement and biological plausibility.

### 4.5 Conclusion and Policy Implications

Our findings highlight the importance of implementing comprehensive intervention strategies that address both environmental and clinical aspects. First, stricter enforcement of evidence‐based air quality standards should be prioritized in regions showing elevated health risks. Second, routine mental health assessments should be integrated into clinical protocols for managing environmentally associated diseases. Additionally, develop gut‐liver‐brain axis biomarkers to monitor intervention efficacy. By dismantling the “pollution‐depression‐disease” triad through such multisectoral strategies, we may mitigate the accelerating burden of environmentally driven digestive diseases.

## 5 Conclusion

This study pioneers the identification of depression as a mediator linking air pollution to GI/liver diseases in a nationally representative cohort. The findings advocate for integrative policies targeting air quality improvement and mental health interventions to alleviate the dual burden of environmental and psychological morbidity.

## Author Contributions

Yanqi Kou, Shicai Ye, Yuping Yang, and Mouji Liang contributed to conceptualization, methodology, formal analysis, investigation, writing – original draft. Yanqi Kou, Yuping Yang, Lei Ge, Ling Qin, Chunyi Wu, Liping Zhan, Ke Yang, and Zhuoyan Lu contributed to investigation, data curation, validation, resources. Yanqi Kou, Yanping Ha, Yuan Tian, Botao Luo, and Yuping Yang contributed to supervision, project administration, funding acquisition, writing – review and editing.

## Funding

This study was supported by the In‐Hospital Funding Clinical Research Project of the Affiliated Hospital of Guangdong Medical University (No. LCYJ2023A002), the Guangdong Provincial Medical Science and Technology Research Fund Project (No. B2024202), and the Clinical Research Special Fund Project of the Guangdong Medical Association (No. 2024QC‐A2004).

## Disclosure

Declaration of Figures Authenticity: All figures submitted have been created by the authors who confirm that the images are original with no duplication and have not been previously published in whole or in part.

## Ethics Statement

The original CHARLS was reviewed and approved by the Biomedical Ethics Committee of Peking University (Approval No. IRB00001052‐11015). All participants in the CHARLS baseline survey provided written informed consent. This study is a secondary analysis of the de‐identified data from CHARLS and the aggregated, publicly available data from the GBD Study. As no primary data collection was involved and all data are anonymized, the current analysis was exempt from additional ethical approval by institutional review.

## Conflicts of Interest

The authors declare no conflicts of interest.

## Supporting information


**Figure S1:** Associations between air pollution and digestive diseases using GBD 2023 data.
**Table S1:** Number and percentage of missing values for each variable in the cohort.
**Table S2:** Spearman correlation coefficients among air pollutants.
**Table S3:** Associations between air pollutants and GI/liver diseases in single‐and two‐pollutant models.
**Table S4:** Association between CESD‐10 and GI and liver diseases.
**Table S5:** Mediation analysis for the associations between air pollution and GI and liver diseases.
**Table S6:** Mediation analysis of the associations between air pollution and GI and liver diseases using the pre‐event CESD‐10 score.
**Table S7:** Characteristics of study patients before and after PSM.
**Table S8:** Sensitivity analysis of using propensity score matching in the association between air pollution and GI and liver diseases.
**Table S9:** Sensitivity analysis of the association between air pollution and GI and liver diseases using continuous variables of air pollutants.
**Table S10:** Sensitivity analysis by excluding participants in poor/very poor health in the association between air pollution and GI and liver diseases.
**Table S11:** Associations between air pollution and GI diseases.
**Table S12:** Associations between air pollution and liver diseases.
**Table S13:** Associations between air pollution and GI/liver diseases using complete‐case analysis.

## Data Availability

The data that support the findings of this study are openly available in CHARLS at https://charls.pku.edu.cn/ and GBD Results Tool (https://vizhub.healthdata.org/gbd‐results/).
