# Supplementary material for: Depression Mediates the Association Between Ambient Air Pollution and Gastrointestinal/Liver Diseases: A Prospective Cohort Study
Source: CNS Neurosci Ther. 2026 Apr 16;32(4):e70878. doi: 10.1002/cns.70878 (PMC13087092; doi:10.1002/cns.70878)
Supplement: Supplementary file 1 — Figure S1: Associations between air pollution and digestive diseases using GBD 2023 data. Table S1: Number and percentage of missing values for each variable in the cohort. Table S2: Spearman correlation coefficients among air pollutants. Table S3: Associations between air pollutants and GI/liver diseases in single‐and two‐pollutant models. Table S4: Association between CESD‐10 and GI and liver diseases. Table S5: Mediation analysis for the associations between air pollution and GI and liver diseases. Table S6: Mediation analysis of the associations between air pollution and GI and liver diseases using the pre‐event CESD‐10 score. Table S7: Characteristics of study patients before and after PSM. Table S8: Sensitivity analysis of using propensity score matching in the association between air pollution and GI and liver diseases. Table S9: Sensitivity analysis of the association between air pollution and GI and liver diseases using continuous variables of air pollutants. Table S10: Sensitivity analysis by excluding participants in poor/very poor health in the association between air pollution and GI and liver diseases. Table S11: Associations between air pollution and GI diseases. Table S12: Associations between air pollution and liver diseases. Table S13: Associations between air pollution and GI/liver diseases using complete‐case analysis. [file CNS-32-e70878-s001.docx]

**Supplementary Material**

**Table of Contents**

[**Figure S1. Associations Between Air Pollution and Digestive Diseases Using GBD 2023 Data** 2](#_Toc216610649)

[**Table S1. Number and percentage of missing values for each variable in the cohort.** 3](#_Toc216610650)

[**Table S2. Spearman correlation coefficients among air pollutants** 3](#_Toc216610651)

[**Table S3. Associations between air pollutants and GI/liver diseases in single-and two-pollutant models** 4](#_Toc216610652)

[**Table S4. Association between CESD-10 and GI and liver diseases.** 5](#_Toc216610653)

[**Table S5. Mediation analysis for the associations between air pollution and GI and liver diseases.** 5](#_Toc216610654)

[**Table S6. Mediation analysis of the associations between air pollution and GI and liver diseases using the pre-event CESD-10 score.** 6](#_Toc216610655)

[**Table S7. Characteristics of study patients before and after PSM** 7](#_Toc216610656)

[**Table S8. Sensitivity analysis of using propensity score matching in the association between air pollution and GI and liver diseases** 9](#_Toc216610657)

[**Table S9. Sensitivity analysis of the association between air pollution and GI and liver diseases using continuous variables of air pollutants** 10](#_Toc216610658)

[**Table S10. Sensitivity analysis by excluding participants who in poor/very poor health in the association between air pollution and GI and liver diseases** 10](#_Toc216610659)

[**Table S11. Associations between air pollution and GI diseases** 11](#_Toc216610660)

[**Table S12. Associations between air pollution and liver diseases** 11](#_Toc216610661)

[**Table S13. Associations between air pollution and GI/liver diseases using complete-case analysis.** 12](#_Toc216610662)

**Figure S1. Associations Between Air Pollution and Digestive Diseases Using GBD 2023 Data**


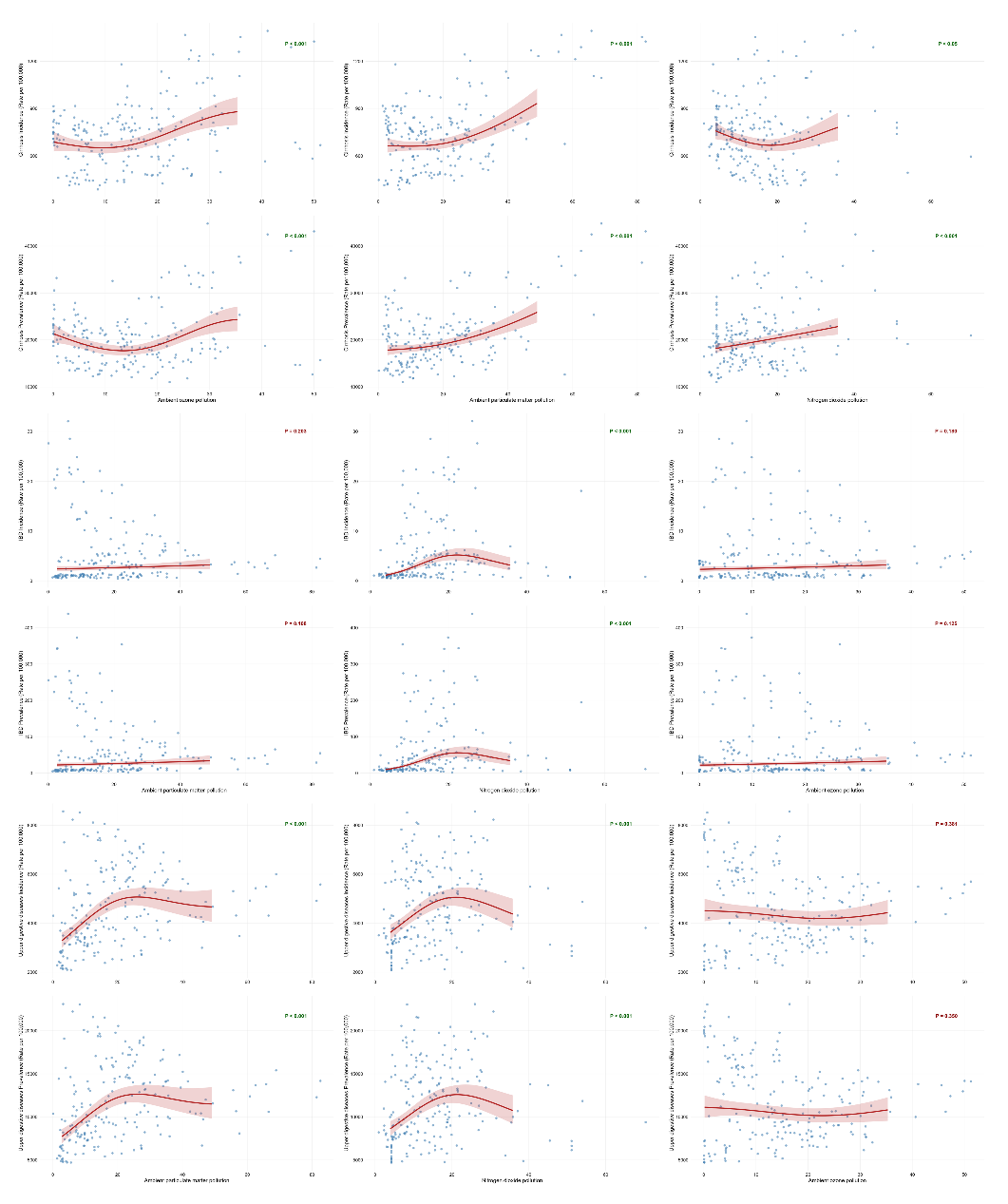


**Table S1. Number and percentage of missing values for each variable in the cohort.**

| **Variable** | **N (%) Missing** |
| --- | --- |
| Age | 47 (0.3%) |
| BMI | 2741 (14.6%) |
| CESD-10 | 1352 (7.2%) |
| Region | 0 (0.0%) |
| Sex | 0 (0.0%) |
| Residence | 0 (0.0%) |
| Marital status | 11 (0.1%) |
| Health status | 340 (1.8%) |
| Smoking | 55 (0.3%) |
| Drinking | 57 (0.3%) |
| Education level | 34 (0.2%) |
| PA | 929 (5.0%) |
| Retirement status | 188 (1.0%) |

Abbreviations: BMI, body mass index (calculated as weight in kilograms divided by height in meters squared); CESD-10, Center for Epidemiological Studies Depression Scale-10; PA, physical activity.

**Table S2. Spearman correlation coefficients among air pollutants**

|  | **PM_1_** | **PM_2.5_** | **PM_10_** | **SO_2_** | **CO** | **O_3_** | **NO_2_** |  |
| --- | --- | --- | --- | --- | --- | --- | --- | --- |
| **PM_1_** | 1 |  |  |  |  |  |  |  |
| **PM_2.5_** | 0.95 | 1 |  |  |  |  |  |  |
| **PM_10_** | 0.87 | 0.89 | 1 |  |  |  |  |  |
| **SO_2_** | 0.65 | 0.65 | 0.74 | 1 |  |  |  |  |
| **CO** | 0.60 | 0.61 | 0.65 | 0.71 | 1 |  |  |  |
| **O_3_** | | 0.52 | 0.51 | 0.58 | 0.38 | 0.35 | 1 |  |
| **NO_2_** | 0.83 | 0.79 | 0.77 | 0.63 | 0.57 | 0.62 | 1 |  |

Abbreviations:PM_1_, particle with aerodynamic diameter ≤1μm; PM_2.5_, particle with aerodynamic diameter ≤2.5 μm; PM_10_, particle with aerodynamic diameter ≤10 μm; SO_2_, sulfur dioxide; NO_2_, nitrogen dioxide; O_3_, ozone; CO, carbon monoxide; all *P*<0.001.

**Table S3. Associations between air pollutants and GI/liver diseases in single-and two-pollutant models**

| **Air pollutants** | **Single-pollutant model** | | | **Adjusted for PM_1_** | | | **Adjusted for PM_2.5_** | | | **Adjusted for PM_10_** | | | **Adjusted for SO_2_** | | | **Adjusted for CO** | | | **Adjusted for O_3_** | | | **Adjusted for NO_2_** | | |
| --- | --- | --- | --- | --- | --- | --- | --- | --- | --- | --- | --- | --- | --- | --- | --- | --- | --- | --- | --- | --- | --- | --- | --- | --- |
|  | **HR** | **95% CI** | ***P*** | **HR** | **95% CI** | ***P*** | **HR** | **95% CI** | ***P*** | **HR** | **95% CI** | ***P*** | **HR** | **95% CI** | ***P*** | **HR** | **95% CI** | ***P*** | **HR** | **95% CI** | ***P*** | **HR** | **95% CI** | ***P*** |
| **PM_1_** | 1.33 | 1.30, 1.37 | <0.001 | - | - | - | 1.39 | 1.27, 1.52 | <0.001 | 1.26 | 1.19, 1.34 | <0.001 | 0.96 | 0.93, 0.10 | 0.041 | 1.10 | 1.06, 1.14 | <0.001 | 1.69 | 1.63, 1.74 | <0.001 | 1.31 | 1.25, 1.37 | <0.001 |
| **PM_2.5_** | 1.30 | 1.26, 1.33 | <0.001 | 0.96 | 0.88, 1.05 | 0.395 | - | - | - | 1.16 | 1.09, 1.23 | <0.001 | 0.94 | 0.90, 0.97 | <0.001 | 1.06 | 1.03, 1.10 | <0.001 | 1.64 | 1.59, 1.69 | <0.001 | 1.23 | 1.18, 1.28 | <0.001 |
| **PM_10_** | 1.29 | 1.25, 1.32 | <0.001 | 1.06 | 1.00, 1.12 | 0.035 | 1.13 | 1.07, 1.20 | <0.001 | - | - | - | 0.76 | 0.73, 0.80 | <0.001 | 1.02 | 0.97, 1.06 | 0.239 | 1.80 | 1.74, 1.86 | <0.001 | 1.21 | 1.16, 1.26 | <0.001 |
| **SO_2_** | 1.64 | 1.60, 1.69 | <0.001 | 1.68 | 1.62, 1.74 | <0.001 | 1.70 | 1.64, 1.76 | <0.001 | 2.02 | 1.93, 2.11 | <0.001 | - | - | - | 1.56 | 1.53, 1.66 | <0.001 | 2.20 | 2.13, 2.28 | <0.001 | 1.77 | 1.71, 1.84 | <0.001 |
| **CO** | 1.47 | 1.43, 1.51 | <0.001 | 1.40 | 1.36, 1.45 | <0.001 | 1.42 | 1.38, 1.47 | <0.001 | 1.45 | 1.40, 1.51 | <0.001 | 1.04 | 1.00, 1.08 | 0.046 | - | - | - | 1.74 | 1.68, 1.79 | <0.001 | 1.47 | 1.42, 1.52 | <0.001 |
| **O_3_** | 0.77 | 0.74, 0.79 | <0.001 | 0.57 | 0.55, 0.59 | <0.001 | 0.57 | 0.55, 0.59 | <0.001 | 0.51 | 0.49, 0.53 | <0.001 | 0.50 | 0.48, 0.52 | <0.001 | 0.61 | 0.59, 0.63 | <0.001 | - | - | - | 0.51 | 0.49, 0.53 | <0.001 |
| **NO_2_** | 1.27 | 1.23, 1.30 | <0.001 | 1.03 | 0.98, 1.08 | 0.253 | 1.08 | 1.04, 1.13 | <0.001 | 1.09 | 1.04, 1.14 | <0.001 | 0.88 | 0.85, 0.91 | <0.001 | 1.00 | 0.97, 1.04 | 0.928 | 1.80 | 1.74, 1.87 | <0.001 | - | - | - |
| Abbreviations:PM_1_, particle with aerodynamic diameter ≤1μm; PM_2.5_, particle with aerodynamic diameter ≤2.5 μm; PM_10_, particle with aerodynamic diameter ≤10 μm; SO_2_, [sulfur dioxide](https://www.sciencedirect.com/topics/earth-and-planetary-sciences/sulphur-dioxide); NO_2_, [nitrogen dioxide](https://www.sciencedirect.com/topics/earth-and-planetary-sciences/nitrogen-dioxide); O_3_, ozone; CO, carbon monoxide; HR, Hazard Ratio; CI, Confidence Interval. Model: adjusted for region, sex, age, residence, marital status, health status, BMI, smoking, drinking, education level, retirement status, and PA. | | | | | | | | | | | | | | | | | | | | | | | | |

**Table S4. Association between CESD-10 and GI and liver diseases.**

| **Characteristic** | **Model 1** | | | | **Model 2** | | | **Model 3** | | |
| --- | --- | --- | --- | --- | --- | --- | --- | --- | --- | --- |
|  | **HR** | **95% CI** | ***P*** | **HR** | | **95% CI** | ***P*** | **HR** | **95% CI** | ***P*** |
| **CESD-10 (continuous)** | 1.07 | 1.07, 1.07 | <0.001 | 1.07 | | 1.07, 1.08 | <0.001 | 1.06 | 1.06, 1.06 | <0.001 |
| **CESD-10** |  |  |  |  | |  |  |  |  |  |
| [0.00 –3.00] | — | — |  | — | | — |  | — | — |  |
| [3.00 – 6.29] | 1.46 | 1.32, 1.62 | <0.001 | 1.48 | | 1.33, 1.64 | <0.001 | 1.41 | 1.27, 1.57 | <0.001 |
| [6.29 – 11.00] | 2.17 | 1.96, 2.40 | <0.001 | 2.20 | | 1.99, 2.44 | <0.001 | 1.96 | 1.77, 2.18 | <0.001 |
| [11.00 – 30.00] | 3.36 | 3.05, 3.70 | <0.001 | 3.44 | | 3.12, 3.79 | <0.001 | 2.84 | 2.57, 3.15 | <0.001 |

Abbreviations: GI, gastrointestinal; CESD-10, Center for Epidemiological Studies Depression Scale-10; HR, Hazard Ratio; CI, Confidence Interval.

Model 1: no covariates were adjusted
Model 2: adjusted for sex and age
Model 3: adjusted for region, sex, age, residence, marital status, health status, BMI, smoking, drinking, education level, retirement status

**Table S5. Mediation analysis for the associations between air pollution and GI and liver diseases.**

| **Independent variable** | **Mediator** | **Indirect effect** | | **Direct effect** | | **Proportion mediated, % (95% CI)** |
| --- | --- | --- | --- | --- | --- | --- |
|  |  | **Coefficient (95% CI)** | ***P*** | **Coefficient (95% CI)** | ***P*** |  |
| **PM_1_** | CESD-10 | 0.00161 (0.00072, 0.00265) | <0.001 | 0.03159 (0.02749, 0.03478) | <0.001 | 4.7 (2.3, 8.1) |
| **PM_2.5_** | CESD-10 | 0.00167 (0.00076, 0.00272) | <0.001 | 0.02965 (0.02540, 0.03294) | <0.001 | 5.1 (2.6, 8.9) |
| **PM_10_** | CESD-10 | 0.00068 (-0.00027, 0.00175) | 0.220 | 0.02909 (0.02475, 0.03250) | <0.001 | 2.2 (-1.0, 6.1) |
| **SO_2_** | CESD-10 | 0.00210 (0.00140, 0.00295) | <0.001 | 0.04884 (0.04645, 0.05076) | <0.001 | 4.0 (2.8, 5.7) |
| **CO** | CESD-10 | 0.00209 (0.00131, 0.00305) | <0.001 | 0.04166 (0.03857, 0.04418) | <0.001 | 4.7 (3.0, 7.0) |
| **O_3_** | CESD-10 | -0.00459 (-0.00599, -0.00319) | <0.001 | -0.03627 (-0.04354, -0.03028) | <0.001 | 11.3 (7.4, 14.5) |
| **NO_2_** | CESD-10 | 0.00138 (0.00045, 0.00247) | <0.001 | 0.02671 (0.02220, 0.03026) | <0.001 | 4.7 (1.7, 9.0) |

Abbreviations: PM_1_, particle with aerodynamic diameter ≤1μm; PM_2.5_, particle with aerodynamic diameter ≤2.5 μm; PM_10_, particle with aerodynamic diameter ≤10 μm; SO_2_, [sulfur dioxide](https://www.sciencedirect.com/topics/earth-and-planetary-sciences/sulphur-dioxide); NO_2_, [nitrogen dioxide](https://www.sciencedirect.com/topics/earth-and-planetary-sciences/nitrogen-dioxide); O_3_, ozone; CO, carbon monoxide; HR, Hazard Ratio; CI, Confidence Interval; CESD-10, Center for Epidemiological Studies Depression Scale-10.

**Table S6. Mediation analysis of the associations between air pollution and GI and liver diseases using the pre-event CESD-10 score.**

| **Independent variable** | **Mediator** | **Indirect effect** | | **Direct effect** | | **Proportion mediated, % (95% CI)** |
| --- | --- | --- | --- | --- | --- | --- |
|  |  | **Coefficient (95% CI)** | ***P*** | **Coefficient (95% CI)** | ***P*** |  |
| **PM_1_** | CESD-10 | 0.00174 (0.00048, 0.00296) | <0.001 | 0.03559 (0.02668, 0.04531) | <0.001 | 3.2 (2.0, 4.9) |
| **PM_2.5_** | CESD-10 | 0.00169 (0.00071, 0.00257) | <0.001 | 0.02911 (0.02511, 0.03325) | <0.001 | 5.5 (2.4, 8.1) |
| **PM_10_** | CESD-10 | 0.00070 (-0.00034, 0.00161) | 0.196 | 0.02854 (0.02447, 0.03280) | <0.001 | 2.5 (-1.4, 5.4) |
| **SO_2_** | CESD-10 | 0.00232 (0.00181, 0.00318) | <0.001 | 0.04857 (0.04594, 0.05007) | <0.001 | 4.5 (3.6, 6.4) |
| **CO** | CESD-10 | 0.00212 (0.00127, 0.00289) | <0.001 | 0.04129 (0.03826, 0.04393) | <0.001 | 4.9 (2.9, 6.5) |
| **O_3_** | CESD-10 | -0.00437 (-0.00527, -0.00315) | <0.001 | -0.03711 (-0.04340, -0.03128) | <0.001 | 10.7 (8.0, 12.6) |
| **NO_2_** | CESD-10 | 0.00141 (0.00038, 0.00231) | <0.001 | 0.02614 (0.02192, 0.03054) | <0.001 | 5.2 (1.6, 8.2) |

Abbreviations: PM_1_, particle with aerodynamic diameter ≤1μm; PM_2.5_, particle with aerodynamic diameter ≤2.5 μm; PM_10_, particle with aerodynamic diameter ≤10 μm; SO_2_, [sulfur dioxide](https://www.sciencedirect.com/topics/earth-and-planetary-sciences/sulphur-dioxide); NO_2_, [nitrogen dioxide](https://www.sciencedirect.com/topics/earth-and-planetary-sciences/nitrogen-dioxide); O_3_, ozone; CO, carbon monoxide; HR, Hazard Ratio; CI, Confidence Interval; CESD-10, Center for Epidemiological Studies Depression Scale-10.

**Table S7. Characteristics of study patients before and after PSM**

| **Characteristics** | **Unmatched** | | | **Matched** | | |
| --- | --- | --- | --- | --- | --- | --- |
|  | **Non-GI and liver diseases**  (n = 13,726) | **GI and liver diseases**  (n = 5,029) | ***P*** | **Non-GI and liver diseases**  (n = 4970) | **GI and liver diseases**  (n = 4970) | ***P*** |
| **Age, (years, M ± SD)** | 57 ± 11 | 56 ± 10 | <0.001 | 56 ± 10 | 56 ± 10 | 0.667 |
| **BMI, (kg/m2, M ± SD)** | 24.0 ± 3.7 | 23.9 ± 4.0 | 0.083 | 24.0 ± 3.8 | 24.1 ± 3.6 | 0.838 |
| **CESD-10, (score, M ± SD)** | 7 ± 6 | 10 ± 6 | <0.001 | 10 ± 6 | 10 ± 6 | 0.899 |
| **Region, n (%)** |  |  | 0.453 |  |  | 0.804 |
| Central | 2,039 (15%) | 770 (15%) |  | 745 (15%) | 759 (15%) |  |
| Eastern | 6,252 (46%) | 2,327 (46%) |  | 2,260 (45%) | 2,291 (46%) |  |
| Northeast | 1,037 (8%) | 356 (7%) |  | 372 (7%) | 355 (7%) |  |
| Western | 4,398 (32%) | 1,576 (31%) |  | 1,593 (32%) | 1,565 (31%) |  |
| **Sex, n (%)** |  |  | <0.001 |  |  | 0.629 |
| Female | 6,880 (50%) | 2,283 (45%) |  | 2,241 (45%) | 2,265 (46%) |  |
| Male | 6,846 (50%) | 2,746 (55%) |  | 2,729 (55%) | 2,705 (54%) |  |
| **Residence, n (%)** |  |  | <0.001 |  |  | 0.790 |
| Rural | 7,617 (55%) | 3,027 (60%) |  | 3,005 (60%) | 2,992 (60%) |  |
| Urban | 6,109 (45%) | 2,002 (40%) |  | 1,965 (40%) | 1,978 (40%) |  |
| **Marital status, n (%)** |  |  | 0.138 |  |  | 0.469 |
| Married | 12,233 (89%) | 4,520 (90%) |  | 4,444 (89%) | 4,466 (90%) |  |
| Single | 1,493 (11%) | 509 (10%) |  | 526 (11%) | 504 (10%) |  |
| **Health status, n (%)** |  |  | <0.001 |  |  | 0.897 |
| Fair | 6,717 (49%) | 2,542 (51%) |  | 2,555 (51%) | 2,534 (51%) |  |
| Good/very good | 4,454 (32%) | 963 (19%) |  | 948 (19%) | 963 (19%) |  |
| Poor/very poor | 2,555 (19%) | 1,524 (30%) |  | 1,467 (30%) | 1,473 (30%) |  |
| **Smoking, n (%)** |  |  | 0.004 |  |  | 0.633 |
| No | 8,282 (60%) | 3,152 (63%) |  | 3,131 (63%) | 3,108 (63%) |  |
| Yes | 5,444 (40%) | 1,877 (37%) |  | 1,839 (37%) | 1,862 (37%) |  |
| **Drinking, n (%)** |  |  | 0.167 |  |  | 0.871 |
| No | 7,703 (56%) | 2,879 (57%) |  | 2,853 (57%) | 2,845 (57%) |  |
| Yes | 6,023 (44%) | 2,150 (43%) |  | 2,117 (43%) | 2,125 (43%) |  |
| **Education level, n (%)** |  |  | <0.001 |  |  | 0.547 |
| Elementary school or below | 9,301 (68%) | 3,641 (72%) |  | 3,562 (72%) | 3,589 (72%) |  |
| Middle school or above | 4,425 (32%) | 1,388 (28%) |  | 1,408 (28%) | 1,381 (28%) |  |
| **Retirement status, n (%)** |  |  | 0.009 |  |  | 0.772 |
| No | 12,062 (88%) | 4,489 (89%) |  | 4,425 (89%) | 4,434 (89%) |  |
| Yes | 1,664 (12%) | 540 (11%) |  | 545 (11%) | 536 (11%) |  |

Abbreviations: BMI, body mass index (calculated as weight in kilograms divided by height in meters squared); GI, gastrointestinal; CESD-10, Center for Epidemiological Studies Depression Scale-10; PSM, propensity score matching.

**Table S8. Sensitivity analysis of using propensity score matching in the association between air pollution and GI and liver diseases**

| **Air Pollutants** | **Model 1** | | | **Model 2** | | | **Model 3** | | |  |
| --- | --- | --- | --- | --- | --- | --- | --- | --- | --- | --- |
|  | **HR** | **95% CI** | ***P*** | **HR** | **95% CI** | ***P*** | **HR** | **95% CI** | ***P*** |  |
| **PM_1_** | 1.27 | 1.24, 1.30 | <0.001 | 1.27 | 1.23, 1.30 | <0.001 | 1.28 | 1.24, 1.31 | <0.001 |  |
| **PM_2.5_** | 1.28 | 1.25, 1.31 | <0.001 | 1.28 | 1.25, 1.31 | <0.001 | 1.28 | 1.24, 1.31 | <0.001 |  |
| **PM_10_** | 1.28 | 1.25, 1.31 | <0.001 | 1.28 | 1.25, 1.31 | <0.001 | 1.21 | 1.18, 1.25 | <0.001 |  |
| **SO_2_** | 1.46 | 1.43, 1.50 | <0.001 | 1.46 | 1.43, 1.50 | <0.001 | 1.52 | 1.48, 1.56 | <0.001 |  |
| **CO** | 1.42 | 1.38, 1.46 | <0.001 | 1.42 | 1.38, 1.45 | <0.001 | 1.41 | 1.37, 1.45 | <0.001 |  |
| **O_3_** | | 0.87 | 0.85, 0.89 | <0.001 | 0.86 | 0.84, 0.89 | <0.001 | 0.78 | 0.75, 0.80 | <0.001 |
| **NO_2_** | 1.21 | 1.18, 1.24 | <0.001 | 1.21 | 1.18, 1.24 | <0.001 | 1.25 | 1.21, 1.28 | <0.001 |  |

Abbreviations:PM1, particle with aerodynamic diameter ≤1μm; PM2.5, particle with aerodynamic diameter ≤2.5 μm; PM10, particle with aerodynamic diameter ≤10 μm; SO2, sulfur dioxide; NO2, nitrogen dioxide; O3, ozone; CO, carbon monoxide; HR, Hazard Ratio; CI, Confidence Interval.

Model 1: no covariates were adjusted

Model 2: adjusted for sex and age

Model 3: adjusted for region, sex, age, residence, marital status, health status, BMI, smoking, drinking, education level, retirement status, and CESD-10

**Table S9. Sensitivity analysis of the association between air pollution and GI and liver diseases using continuous variables of air pollutants**

| **Air Pollutants** | **Model 1** | | | **Model 2** | | | **Model 3** | | |  |
| --- | --- | --- | --- | --- | --- | --- | --- | --- | --- | --- |
|  | **HR** | **95% CI** | ***P*** | **HR** | **95% CI** | ***P*** | **HR** | **95% CI** | ***P*** |  |
| **PM_1_** | 1.04 | 1.04, 1.04 | <0.001 | 1.04 | 1.04, 1.04 | <0.001 | 1.05 | 1.04, 1.05 | <0.001 |  |
| **PM_2.5_** | 1.02 | 1.02, 1.02 | <0.001 | 1.02 | 1.02, 1.02 | <0.001 | 1.02 | 1.02, 1.03 | <0.001 |  |
| **PM_10_** | 1.01 | 1.01, 1.01 | <0.001 | 1.01 | 1.01, 1.01 | <0.001 | 1.01 | 1.01, 1.01 | <0.001 |  |
| **SO_2_** | 1.04 | 1.04, 1.04 | <0.001 | 1.04 | 1.04, 1.04 | <0.001 | 1.05 | 1.05, 1.05 | <0.001 |  |
| **CO** | 4.37 | 3.94, 4.84 | <0.001 | 4.36 | 3.93, 4.83 | <0.001 | 4.53 | 4.04, 5.07 | <0.001 |  |
| **O_3_** | | 0.98 | 0.98, 0.98 | <0.001 | 0.98 | 0.97, 0.98 | <0.001 | 0.96 | 0.96, 0.97 | <0.001 |
| **NO_2_** | 1.02 | 1.02, 1.02 | <0.001 | 1.02 | 1.02, 1.02 | <0.001 | 1.02 | 1.02, 1.03 | <0.001 |  |

Abbreviations:PM1, particle with aerodynamic diameter ≤1μm; PM2.5, particle with aerodynamic diameter ≤2.5 μm; PM10, particle with aerodynamic diameter ≤10 μm; SO2, sulfur dioxide; NO2, nitrogen dioxide; O3, ozone; CO, carbon monoxide; HR, Hazard Ratio; CI, Confidence Interval.

Model 1: no covariates were adjusted

Model 2: adjusted for sex and age

Model 3: adjusted for region, sex, age, residence, marital status, health status, BMI, smoking, drinking, education level, retirement status, and CESD-10

**Table S10.** **Sensitivity analysis by excluding participants who in poor/very poor health in the association between air pollution and GI and liver diseases**

| **Air Pollutants** | **Model 1** | | | **Model 2** | | | **Model 3** | | |  |
| --- | --- | --- | --- | --- | --- | --- | --- | --- | --- | --- |
|  | **HR** | **95% CI** | ***P*** | **HR** | **95% CI** | ***P*** | **HR** | **95% CI** | ***P*** |  |
| **PM_1_** | 1.31 | 1.27, 1.35 | <0.001 | 1.31 | 1.27, 1.35 | <0.001 | 1.34 | 1.30, 1.39 | <0.001 |  |
| **PM_2.5_** | 1.31 | 1.27, 1.35 | <0.001 | 1.30 | 1.26, 1.34 | <0.001 | 1.32 | 1.27, 1.36 | <0.001 |  |
| **PM_10_** | 1.30 | 1.26, 1.34 | <0.001 | 1.30 | 1.26, 1.34 | <0.001 | 1.31 | 1.27, 1.35 | <0.001 |  |
| **SO_2_** | 1.56 | 1.51, 1.61 | <0.001 | 1.56 | 1.51, 1.61 | <0.001 | 1.64 | 1.59, 1.70 | <0.001 |  |
| **CO** | 1.47 | 1.42, 1.51 | <0.001 | 1.46 | 1.42, 1.51 | <0.001 | 1.47 | 1.43, 1.52 | <0.001 |  |
| **O_3_** | | 0.86 | 0.84, 0.89 | <0.001 | 0.85 | 0.83, 0.88 | <0.001 | 0.77 | 0.74, 0.80 | <0.001 |
| **NO_2_** | 1.24 | 1.20, 1.28 | <0.001 | 1.23 | 1.20, 1.27 | <0.001 | 1.28 | 1.24, 1.33 | <0.001 |  |
| Abbreviations:PM_1_, particle with aerodynamic diameter ≤1μm; PM_2.5_, particle with aerodynamic diameter ≤2.5 μm; PM_10_, particle with aerodynamic diameter ≤10 μm; SO_2_, [sulfur dioxide](https://www.sciencedirect.com/topics/earth-and-planetary-sciences/sulphur-dioxide); NO_2_, [nitrogen dioxide](https://www.sciencedirect.com/topics/earth-and-planetary-sciences/nitrogen-dioxide); O_3_, ozone; CO, carbon monoxide; HR, Hazard Ratio; CI, Confidence Interval. | | | | | | | | | |  |
| Model 1: no covariates were adjusted  Model 2: adjusted for sex and age  Model 3: adjusted for region, sex, age, residence, marital status, health status, BMI, smoking, drinking, education level, retirement status, and CESD-10 | | | | | | | | | |  |

**Table S11. Associations between air pollution and GI diseases**

| **Air pollutants** | **Model 1** | | | **Model 2** | | | **Model 3** | | |  |
| --- | --- | --- | --- | --- | --- | --- | --- | --- | --- | --- |
|  | **HR** | **95% CI** | ***P*** | **HR** | **95% CI** | ***P*** | **HR** | **95% CI** | ***P*** |  |
| **PM_1_** | 1.24 | 1.20, 1.28 | <0.001 | 1.23 | 1.20, 1.27 | <0.001 | 1.29 | 1.24, 1.33 | <0.001 |  |
| **PM_2.5_** | 1.22 | 1.18, 1.26 | <0.001 | 1.22 | 1.18, 1.25 | <0.001 | 1.25 | 1.20, 1.29 | <0.001 |  |
| **PM_10_** | 1.20 | 1.17, 1.24 | <0.001 | 1.20 | 1.16, 1.24 | <0.001 | 1.22 | 1.18, 1.27 | <0.001 |  |
| **SO_2_** | 1.49 | 1.45, 1.54 | <0.001 | 1.50 | 1.45, 1.54 | <0.001 | 1.55 | 1.50, 1.61 | <0.001 |  |
| **CO** | 1.36 | 1.32, 1.41 | <0.001 | 1.36 | 1.32, 1.41 | <0.001 | 1.41 | 1.36, 1.46 | <0.001 |  |
| **O_3_** | | 0.83 | 0.81, 0.86 | <0.001 | 0.81 | 0.79, 0.84 | <0.001 | 0.74 | 0.71, 0.77 | <0.001 |
| **NO_2_** | 1.19 | 1.15, 1.23 | <0.001 | 1.18 | 1.15, 1.22 | <0.001 | 1.23 | 1.18, 1.27 | <0.001 |  |

Abbreviations:PM1, particle with aerodynamic diameter ≤1μm; PM2.5, particle with aerodynamic diameter ≤2.5 μm; PM10, particle with aerodynamic diameter ≤10 μm; SO2, sulfur dioxide; NO2, nitrogen dioxide; O3, ozone; CO, carbon monoxide; HR, Hazard Ratio; CI, Confidence Interval.

Model 1: no covariates were adjusted

Model 2: adjusted for sex and age

Model 3: adjusted for region, sex, age, residence, marital status, health status, BMI, smoking, drinking, education level, retirement status, and PA

**Table S12. Associations between air pollution and liver diseases**

| **Air pollutants** | **Model 1** | | | **Model 2** | | | **Model 3** | | |  |
| --- | --- | --- | --- | --- | --- | --- | --- | --- | --- | --- |
|  | **HR** | **95% CI** | ***P*** | **HR** | **95% CI** | ***P*** | **HR** | **95% CI** | ***P*** |  |
| **PM_1_** | 1.30 | 1.23, 1.37 | <0.001 | 1.30 | 1.23, 1.37 | <0.001 | 1.31 | 1.23, 1.40 | <0.001 |  |
| **PM_2.5_** | 1.29 | 1.22, 1.36 | <0.001 | 1.29 | 1.26, 1.36 | <0.001 | 1.28 | 1.20, 1.36 | <0.001 |  |
| **PM_10_** | 1.32 | 1.25, 1.39 | <0.001 | 1.32 | 1.25, 1.39 | <0.001 | 1.31 | 1.23, 1.40 | <0.001 |  |
| **SO_2_** | 1.62 | 1.53, 1.71 | <0.001 | 1.62 | 1.53, 1.71 | <0.001 | 1.69 | 1.59, 1.80 | <0.001 |  |
| **CO** | 1.47 | 1.39, 1.55 | <0.001 | 1.47 | 1.39, 1.55 | <0.001 | 1.47 | 1.39, 1.56 | <0.001 |  |
| **O_3_** | | 0.91 | 0.86, 0.96 | <0.001 | 0.90 | 0.86, 0.96 | <0.001 | 0.83 | 0.77, 0.88 | <0.001 |
| **NO_2_** | 1.25 | 1.19, 1.33 | <0.001 | 1.25 | 1.19, 1.32 | <0.001 | 1.30 | 1.22, 1.38 | <0.001 |  |

Abbreviations:PM1, particle with aerodynamic diameter ≤1μm; PM2.5, particle with aerodynamic diameter ≤2.5 μm; PM10, particle with aerodynamic diameter ≤10 μm; SO2, sulfur dioxide; NO2, nitrogen dioxide; O3, ozone; CO, carbon monoxide; HR, Hazard Ratio; CI, Confidence Interval.

Model 1: no covariates were adjusted

Model 2: adjusted for sex and age

Model 3: adjusted for region, sex, age, residence, marital status, health status, BMI, smoking, drinking, education level, retirement status, and PA

**Table S13. Associations between air pollution and GI/liver diseases using complete-case analysis.**

| **Air pollutants** | **Model 1** | | | **Model 2** | | | **Model 3** | | |  |
| --- | --- | --- | --- | --- | --- | --- | --- | --- | --- | --- |
|  | **HR** | **95% CI** | ***P*** | **HR** | **95% CI** | ***P*** | **HR** | **95% CI** | ***P*** |  |
| **PM_1_** | 1.31 | 1.28, 1.34 | <0.001 | 1.31 | 1.27, 1.34 | <0.001 | 1.37 | 1.33, 1.41 | <0.001 |  |
| **PM_2.5_** | 1.29 | 1.26, 1.33 | <0.001 | 1.29 | 1.26, 1.32 | <0.001 | 1.32 | 1.28, 1.36 | <0.001 |  |
| **PM_10_** | 1.29 | 1.26, 1.32 | <0.001 | 1.29 | 1.25, 1.32 | <0.001 | 1.29 | 1.26, 1.33 | <0.001 |  |
| **SO_2_** | 1.56 | 1.52, 1.61 | <0.001 | 1.56 | 1.52, 1.61 | <0.001 | 1.68 | 1.63, 1.74 | <0.001 |  |
| **CO** | 1.46 | 1.43, 1.50 | <0.001 | 1.46 | 1.42, 1.50 | <0.001 | 1.50 | 1.46, 1.55 | <0.001 |  |
| **O_3_** | | 0.86 | 0.84, 0.88 | <0.001 | 0.85 | 0.83, 0.87 | <0.001 | 0.74 | 0.71, 0.76 | <0.001 |
| **NO_2_** | 1.23 | 1.20, 1.26 | <0.001 | 1.23 | 1.19, 1.26 | <0.001 | 1.28 | 1.24, 1.33 | <0.001 |  |

Abbreviations:PM1, particle with aerodynamic diameter ≤1μm; PM2.5, particle with aerodynamic diameter ≤2.5 μm; PM10, particle with aerodynamic diameter ≤10 μm; SO2, sulfur dioxide; NO2, nitrogen dioxide; O3, ozone; CO, carbon monoxide; HR, Hazard Ratio; CI, Confidence Interval.

Model 1: no covariates were adjusted

Model 2: adjusted for sex and age

Model 3: adjusted for region, sex, age, residence, marital status, health status, BMI, smoking, drinking, education level, retirement status, and PA
